# Asylum Superintendents

**Published:** 1890-12

**Authors:** 


**The Asylum Superintendency.**—With each change of administration there is a hubbub of excitement created throughout the State with regard to the changes sure or almost sure to be made in the management of the several asylums of the State.

And this excitement is not confined to the medical profession alone, but it extends to and exerts a baneful influence upon the more intelligent inmates of the asylums themselves. From the time a new governor is elected until after the appointment is made these inmates—who, perhaps, by long association have become strongly attached to the medical officers of the asylum, are in a state of vague fear and apprehension that a new man, and, perhaps, an uncongenial one, will soon be assigned to the charge of them—body and soul—and it may well be imagined such fear is *not much* calculated to hasten the recovery of their mental equilibrium. A case in point now: There is a vague rumor that Dr. Wallace—the designer, and, you may say, the supervising architect of the building of the insane asylum at Terrell—and who has had charge of the asylum from its founding, in 1883, is to be removed for political spite or revenge. Just how such rumor started, it is hard to tell, but it has spread, and the inmates of the asylum are in a flutter of excitement, not unmixed with indignation at the prospect of being deprived of the efficient —they say—fatherly care of their long-time superintendent.

Now, the JOURNAL does not believe that J. S. Hogg is built that way; he has too much sense, and too much soul to sacrifice the welfare of some hundreds of this class of unfortunates to a miserable little political pique or spite. Suppose Wallace was *not* a Hogg man—as has been charged—Governor Hogg was elected by such an overwhelming majority he can afford to laugh now at any man's feeble opposition. And suppose *there are*, as alleged, as competent men in the State as Dr. Wallace,—which we doubt very much as to this particular,—is the change justifiable? Dr. Wallace's management of this asylum these many years has been characterized not only by efficiency and economy, but by peace and harmony, (which cannot be said of some others); and his report shows results seldom attained in institutions of this character. We shall have more to say of this subject at some future time.

